# Experimental Study on the Fatigue Degradation of Prestressed Concrete Slabs for Composite Bridges

**DOI:** 10.3390/ma18214878

**Published:** 2025-10-24

**Authors:** Wenjun Li, Rujin Ma, Yuqing Liu, Chen Liang

**Affiliations:** Department of Bridge Engineering, Tongji University, Shanghai 200092, China; 2110361@tongji.edu.cn (W.L.); rjma@tongji.edu.cn (R.M.); yql@tongji.edu.cn (Y.L.)

**Keywords:** composite bridge, prestressed concrete slabs, fatigue test, performance enhancement, evaluation method

## Abstract

Concrete slabs in composite bridges are inevitably subjected to heavy vehicular loads during their service life. To evaluate the fatigue performance of the prestressed concrete slabs in composite bridges, two full-scaled models of prestressed concrete slabs were first designed and tested, with the load amplitude was selected as the variable. To simulate the damage caused by the initial passage of heavy vehicles, this was simplified into the form of a static cyclic load. The mechanical deformation state and crack distribution of the slab were analyzed. Further, a finite-element model was established, and a parametric analysis based on the variation in loading form, such as monotonic displacement loading, static cyclic loading followed by monotonic displacement loading, and cyclic displacement loading, was conducted to discuss the performance-enhancement mechanism of prestressed concrete slabs. Finally, in consideration of the influence of static cyclic damage on the fatigue performance of prestressed concrete slabs, evaluation parameters were proposed to account for static cyclic damage by considering the effects of stresses in concrete, tensile rebar, prestressed tendons, and external loading. A comprehensive fatigue performance evaluation method for prestressed concrete slabs, which neglects the tensile hardening behavior of cracked concrete in the tension zone, was established and verified by test results. The results indicate that the damage caused by static cyclic loading has a significant influence on the fatigue performance of the slab. Applying prestress can significantly mitigate the influence of initial damage on the mechanical and deformation behavior of the slab, which benefits from the prestress compensating for the cracking stress at the bottom of the slab. The proposed fatigue performance-evaluation method for prestressed concrete slabs, which considers static cyclic damage, can predict fatigue deformation behavior with an error of less than 10%, while reasonably determining the fatigue life and failure modes of prestressed concrete slabs. The parametric analysis reveals that when the prestress value exceeds 9 MPa, the failure mode of the prestressed concrete slab transfers from rebar fracture to concrete failure.

## 1. Introduction

During the service life of composite bridges, the concrete slab is directly subjected to the repeated vehicular loads and is susceptible to cracking and fatigue failure under long-term action. Around the 1970s, many concrete slabs were designed considering the beneficial effects of the “arching action”, resulting in a relatively small thickness. Those slabs were soon found to fail due to fatigue, since the arching action has very limited benefits on fatigue behavior [1]. Due to the complex nature of the problem and the uncertainty of the traffic load [2], although current specifications [3,4] provide a design method based on S-N curves, extensive fatigue cracks were still observed in composite bridges [5,6,7]. The moving vehicles and stationary vehicles on the bridge slab influence its dynamic response and lead to potentially hazardous global and local resonance phenomena, resulting in structural damage [8,9]. The trend of a larger transverse span for the bridge slab [10], and fewer main girders in composite girder bridges, has exacerbated the above issues.

Applying prestress is an effective method to enhance the performance of concrete slabs, with an augmentation in flexural strength, punch shear resistance, and fatigue strength without increasing the thickness. Lantsoght et al. [11] demonstrated through reciprocating loading tests that the application of prestress significantly enhances the fatigue punch shear resistance of bridge slabs. Bae et al. [12] investigated the static and fatigue performance of prestressed UHPC slabs and concluded that the performance of slabs satisfied the requirements for cable-stayed bridges, and the application of prestress alters the original failure mode of the bridge slabs [13]. Furthermore, prestress can also be applied through the utilization of BFRP shells [14,15] and CFRP strips [16]. In addition to the bridge sector, the railway industry has increasingly adopted prestressed reinforced concrete ballastless tracks to meet the transportation requirements of heavy-haul trains [17]. The above existing studies focused on directly applying fatigue loads. However, concrete slabs are inevitably subjected to heavy vehicular loads during their service life, which causes damage to the concrete structure, and the influence of damage on the fatigue performance of the concrete structure was assessed using a pseudo-cracking approach [18,19,20].

The fatigue performance evaluation of bridge slabs can be conducted with the aid of the S-N curve in the specification, whereby rationalized numerical modeling enables the quantification of stress amplitude in tensile rebar [21], ultimately permitting the prediction of concrete structural fatigue life. Several scholars conducted extensive fatigue tests on bridge slabs and derived the fatigue-life prediction equation based on the test results [10,22]. Furthermore, Zanuy et al. [23] and Du et al. [24] individually characterized the fatigue damage of materials, including concrete, tensile rebar, and prestressed tendons, and developed a fatigue performance-evaluation framework for prestressed concrete (PC) beams to predict the fatigue deformation behavior of the concrete structure, and the framework can incorporate influences such as corrosion [25] and FRP plate strengthening [26]. Additionally, according to the method for calculating the stiffness of partially cracked sections in concrete structures outlined in the Eurocode [3], the cracking state of the section exerts a substantial influence on the deformation behavior of the bridge slabs. As a critical parameter, the deformation of the bridge slab offers valuable insights for its structural design and optimization. Consequently, it is imperative to precisely characterize the cracking state of the bridge slab following heavy vehicular loads.

To solve the above-mentioned issues, two full-scale PC slab specimens were fabricated. A loading protocol involving initial static cyclic loading followed by fatigue loading was employed to simulate the damage of the concrete slab after experiencing heavy vehicular and subsequently bearing conventional vehicular traffic. The fatigue test was conducted with the load amplitude being the variable, and the mid-span deformation, crack distribution, and evolution of strain were analyzed. On this basis, a finite-element model was developed to investigate the influence of prestress on the mechanical deformation behavior of the slabs, elucidating the performance-enhancement mechanism of PC slabs. Meanwhile, evaluation parameters that account for static cyclic damage were proposed, a fatigue performance-evaluation method was established, and its accuracy was validated through test results.

## 2. Experimental Program

### 2.1. Specimen Design

As Table 1 lists, two full-scaled PC specimens were fabricated, labeled as PC-HL and PC-LH, where *F_s_* denotes the maximum static load, *F_min_* signifies the lower limit of the fatigue load, and *F_max_* indicates the upper limit of the fatigue load; *M* stands for one million times. The main variable is the load magnitude. The first and second letters following the hyphen denote the magnitude of the static and fatigue load, and the letter H or L indicates that the magnitude is high or low, respectively.

**Figure 1:**
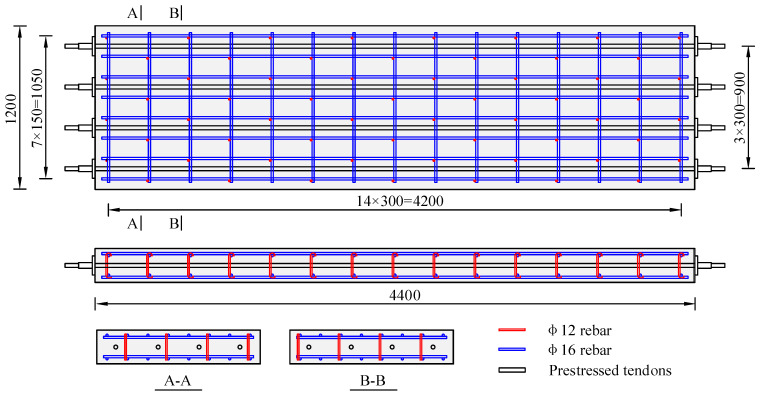
Configurations of test models (unit: mm).

The length, width, and height of the specimens were 4400 mm, 1200 mm, and 250 mm, respectively, as shown in Figure 1. The diameter of the top and bottom rebar was 16 mm. The longitudinal and transverse rebar spacings of the specimen were 150 mm and 300 mm, respectively. The thickness of the rebar cover was 30 mm. Additionally, 12 mm diameter erection rebars were placed between the top and bottom rebars. Four prestressed threaded steel bars with a diameter of 32 mm were set at the midsection of the PC slabs to serve as prestressed tendons.

The prestressed tendons were threaded into the prestressed duct before pouring concrete. Strain gauges were attached to the surface of the prestressed tendons to monitor and control the tensioning stress. The design specifies a prestress of 4 MPa for the PC slab, with a designed tensioning stress of 373 MPa for each prestressed tendon.

### 2.2. Material Properties

In the experiment, rebars with a nominal yield stress of 400 MPa were employed and the material properties were determined through direct tensile test. Concrete with a nominal cubic compressive strength of 50 MPa was used and was tested with five cubic specimens measuring 150 mm × 150 mm × 150 mm and ten cylindrical specimens with dimensions φ150 mm × 300 mm. The material properties of the rebar and concrete are consistent with those reported in previous works [27].

### 2.3. Loading Setup and Measurements

**Figure 2:**
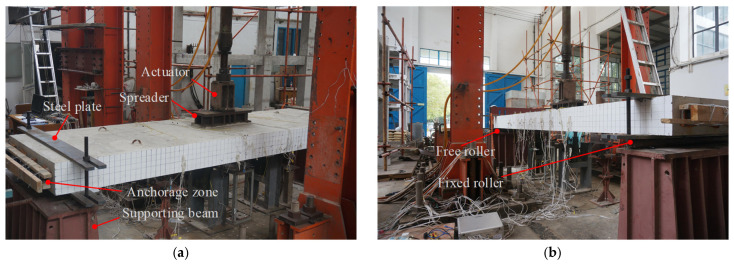
Test device: (**a**) top of the specimen; (**b**) bottom of the specimen.

The loading setup and measurements were identical for PC slab specimens. A 50 tf hydraulic pulsating fatigue-testing machine was utilized to apply a single-point fatigue load. As illustrated in Figure 2, the load was transmitted via a spreader beam to the mid-span, simulating a single-wheel load. To prevent the local crushing of the concrete, steel plates were installed at the support locations of the specimen. A simply supported boundary condition was employed, with a support span of 4000 mm.

**Figure 3:**
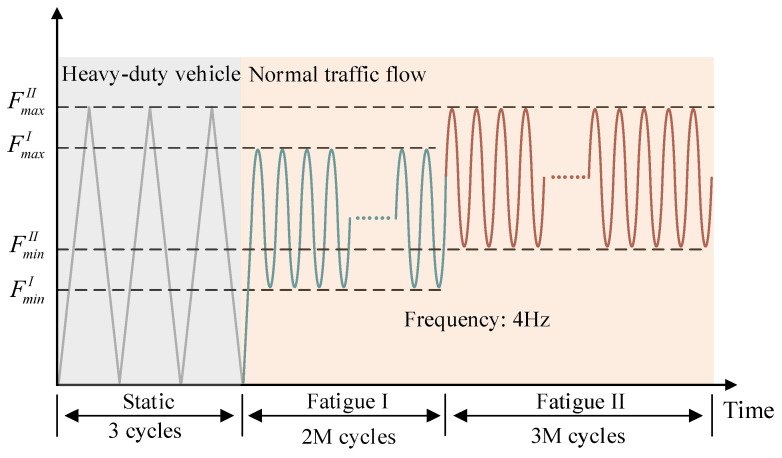
Loading program.

Figure 3 illustrates a simplified schematic of the test loading for various vehicle flows. In this experiment, the status simulated is that in which a heavy vehicle passes first, followed by the regular traffic flow. Specifically, the static cyclic loading was adopted to simulate the damage caused by a heavy vehicle. Lower fatigue loads were used to simulate lighter conventional vehicle traffic, while higher fatigue loads represented heavier conventional vehicle traffic.

The test-loading protocol consists of two phases: static cyclic loading and fatigue loading. Specifically, the static cyclic loading phase emulates the influence of the damage caused by heavy vehicular loads, while the fatigue-loading phase replicates the influence of continuous regular traffic flow. The specific load parameters for each phase are detailed in Table 1. The fatigue load was applied as a sinusoidal waveform at a frequency of 4 Hz. The upper limit of the load (*F_max_*) for the PC slab was determined based on the literature [27], taken as twice the cracking load of the concrete slab, and the load amplitude was determined to represent a single-wheel load of fatigue-load model III in Chinese code JTG D60-2015 [28]. Considering the relatively high flexibility of the specimens, the lower limit of the fatigue load was set at 40% of the upper limit to simulate secondary dead loads such as bridge slab pavement. The static loading consists of three cycles, with the maximum static load determined according to the upper limit of the fatigue load.

**Figure 4:**
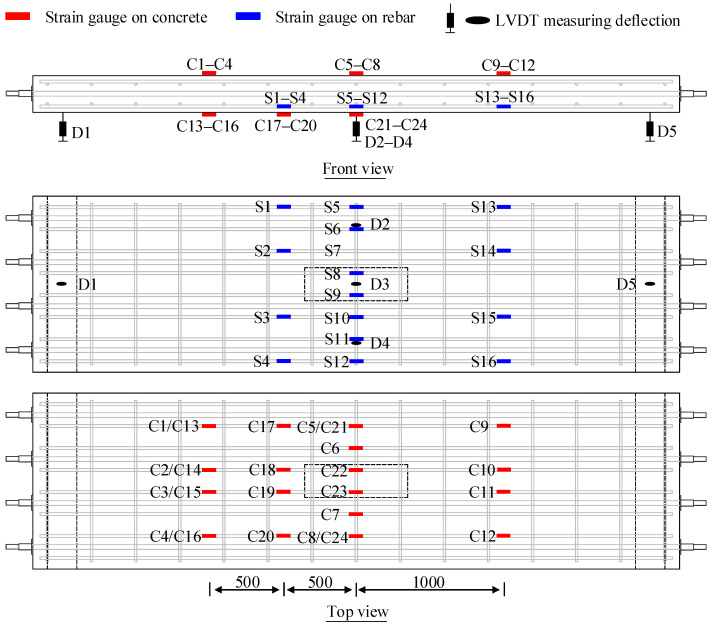
Measurements (unit: mm).

The arrangement of the measurements for the specimens is illustrated in Figure 4. Three linear variable displacement transducers (LVDTs) were positioned at mid-span to measure the deflection. LVDTs were installed at the supports to mitigate the influence of support deformation on the test results. Strain gauges were attached to both the concrete and rebar to monitor strain evolution.

## 3. Experimental Results

### 3.1. Evolution of Displacements

**Figure 5:**
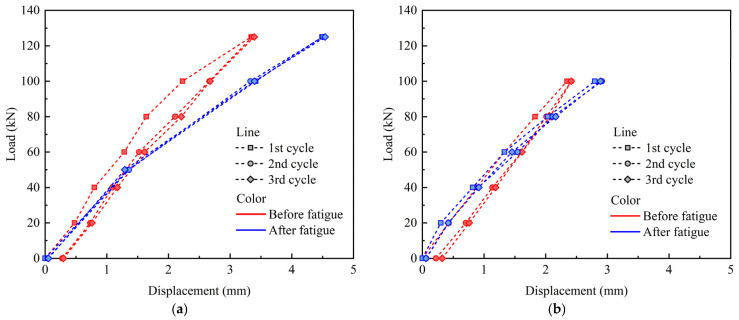
Load–displacement curves under static loading: (**a**) PC-HL; (**b**) PC-LH.

Figure 5 illustrates the variation curve of static cyclic loading and mid-span displacement. The PC slabs remained intact after 5 M fatigue loadings. The deformation evolution of the slabs was further analyzed using static cyclic loading, with identical procedures applied both before and after the fatigue-loading process. Compared to the state before fatigue loading, the residual displacement after fatigue loading was relatively small, suggesting that the deformation of the specimen had entered a stable phase following 5 M load cycles. Furthermore, compared to the conditions prior to fatigue loading, the maximum static displacements of PC-HL and PC-LH increased by 34% and 25%, respectively, after fatigue loading. This suggests that both static and fatigue loading induced significant damage to the PC slabs, with the cumulative damage in PC-HL being greater than that in PC-LH.

**Figure 6:**
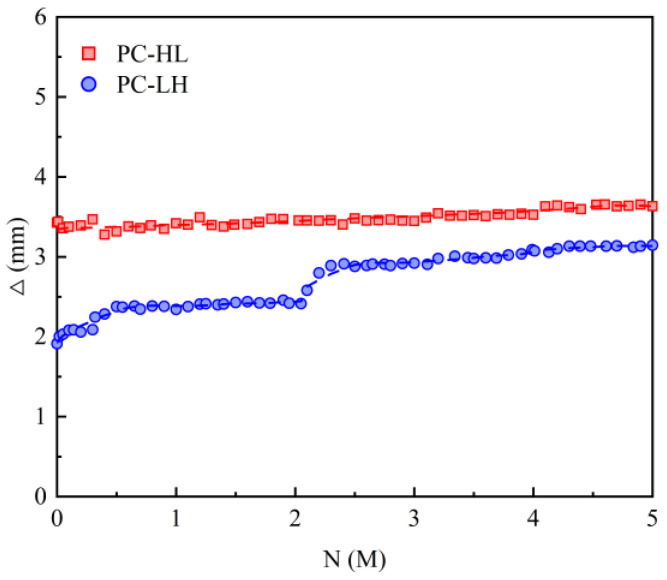
Mid-span displacement amplitude under fatigue loading.

Figure 6 illustrates the variation in mid-span displacement amplitude Δ under fatigue loading. The PC slab did not exhibit fatigue failure after 5 M load cycles, with the displacement amplitude remaining below 3.5 mm. Compared with PC-HL, the specimen PC-LH subjected to low static loading exhibited a smaller fatigue displacement amplitude during the initial stage of fatigue loading. Even when the upper limit of the fatigue load was increased to match the maximum static load of PC-HL, the fatigue displacement amplitude remained relatively small.

### 3.2. Relative Stiffness

**Figure 7:**
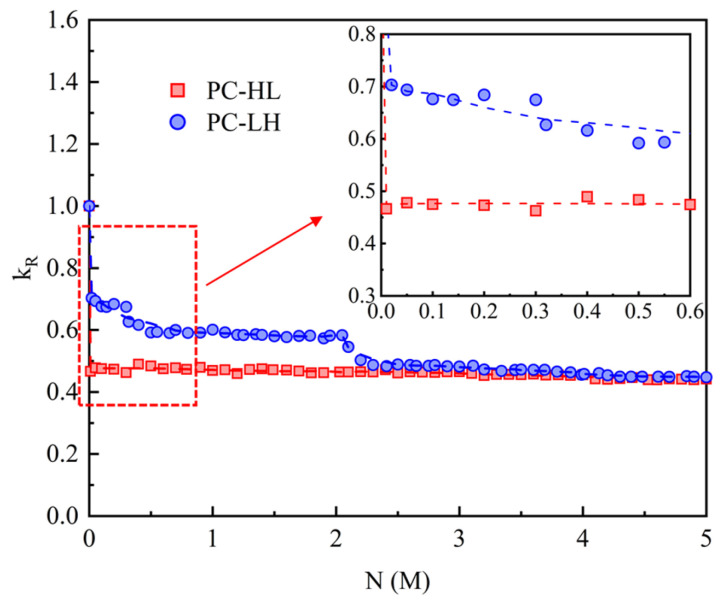
Relative stiffness under fatigue loading.

The relative stiffness *k_R_* was utilized to assess the stiffness degradation process. *k_R_* is defined as the ratio of the secant stiffness after *N* load cycles to that during the first static load cycle. Figure 7 depicts the variation in relative stiffness under fatigue loading. Different static cyclic loading leads to noticeable distinctions in the stiffness degradation process between the specimens PC-HL and PC-LH within 2 M load cycles. However, after increasing the fatigue load for PC-HL, the relative stiffness of PC-HL and PC-LH gradually approached as the number of load cycles increased. After 2.5 M load cycles, the relative stiffness curves of PC-HL and PC-LH exhibited a crossing pattern rather than remaining parallel. This indicates that with further increases in fatigue cycles, the relative stiffness of PC-LH will eventually fall below that of PC-HL, and the greater damage observed in PC-LH was attributed to the higher fatigue-load amplitude.

### 3.3. Crack Distribution

**Figure 8:**
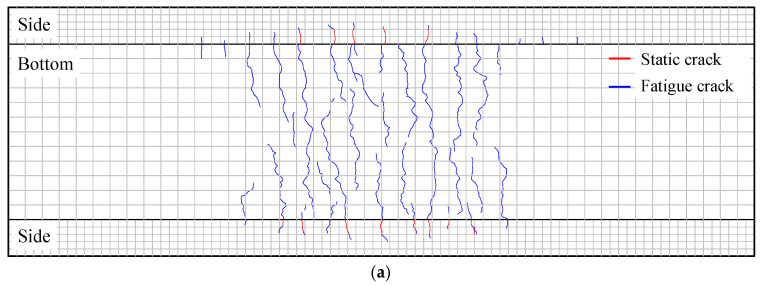

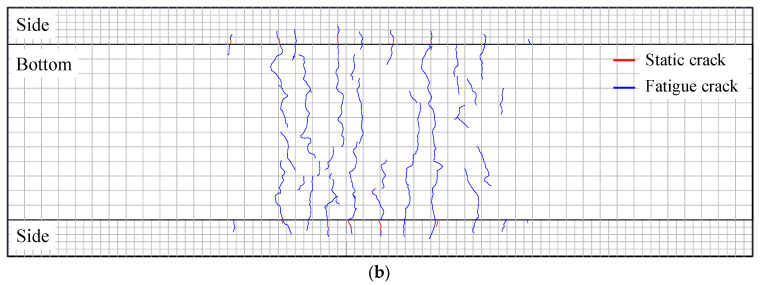
Crack distribution: (**a**) PC-HL; (**b**) PC-LH.

Figure 8 shows the crack distribution after fatigue loading, where static cracks were highlighted in red, and fatigue cracks were indicated in blue. After the completion of static cyclic loading, cracks in the PC slabs were only present on the sides. Specifically, noticeable cracks developed in PC-HL, while the cracks in PC-LH were in the initial stage of formation. After the fatigue test was completed, fewer fatigue cracks were observed at the bottom of the PC slab, and no longitudinal cracks had been detected. Compared with the PC-HL specimens, the PC-LH specimens exhibit a lower propensity for both static and fatigue crack initiation.

**Figure 9:**
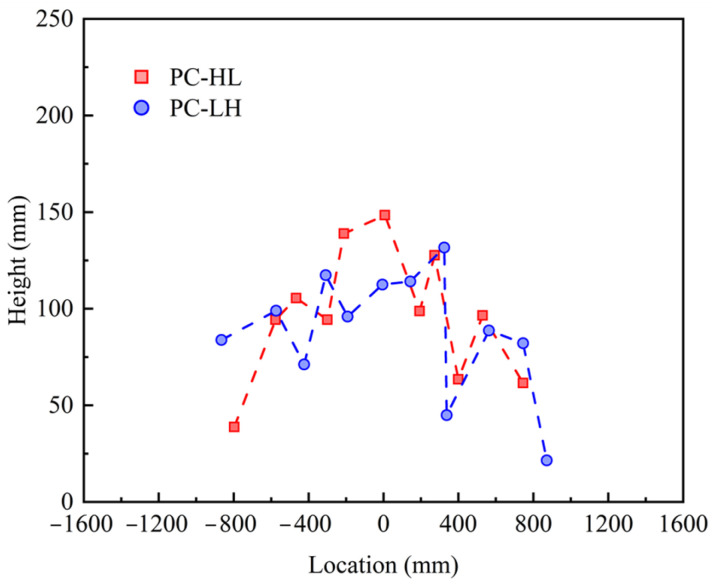
Crack height distribution at the side of the slab.

Figure 9 depicts the crack height distribution of the slab at the side under fatigue loading. The crack distribution range of the PC slab was small, encompassing less than 55% of the effective span length. Furthermore, the crack height in the PC slab was less than 150 mm.

### 3.4. Evolution of Strain

**Figure 10:**
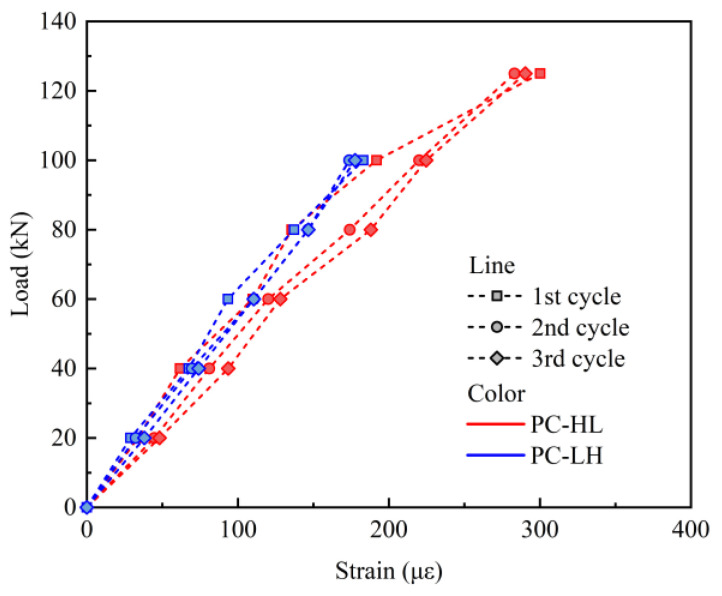
Strain of the top concrete under static loading.

Figure 10 illustrates the strain curves of the top concrete under static cyclic loading. The strain was reset to zero at the beginning of each load cycle. The slope of strain curves for the top concrete of the PC slab exhibited minimal change during the static loading process, and the strain in the bottom concrete remained consistent across various static load cycles, suggesting that the static cyclic loading did not induce significant alterations in the stress distribution within the slab.

**Figure 11:**
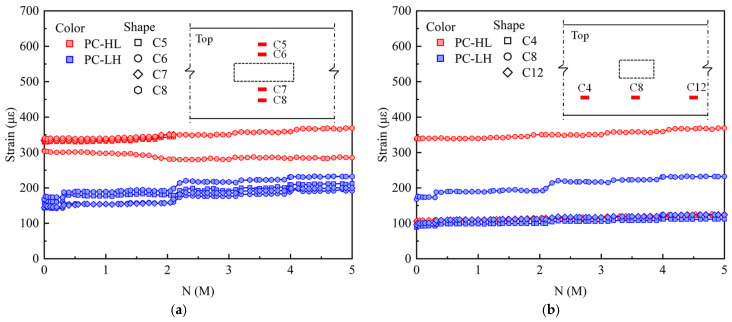
Strain amplitude of top concrete under fatigue loading: (**a**) transverse distribution; (**b**) longitudinal distribution.

Figure 11 shows the variations in strain amplitude of the top concrete under fatigue loading. As shown in Figure 11a, the PC slab exhibited a uniform transverse strain distribution, indicating that the application of prestress significantly enhanced the mechanical performance of the slab. After 2 M load cycles, PC-HL exhibited an increased variation in transverse stress distribution, with higher static loads significantly influencing the stress distribution in the top concrete of the PC slab. Figure 11b depicts that the PC slab exhibited a symmetric longitudinal stress distribution during fatigue loading.

**Figure 12:**
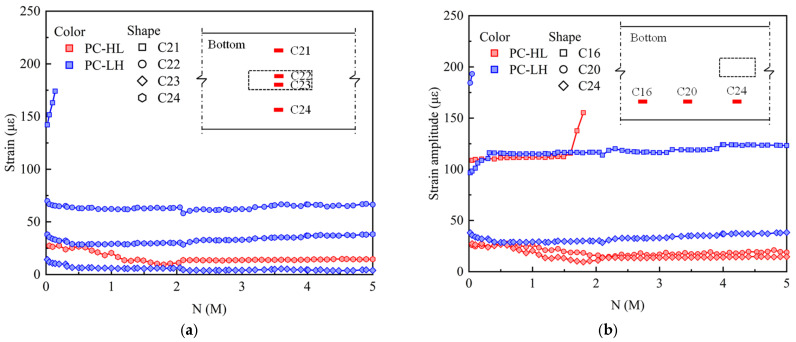
Strain amplitude of bottom concrete under fatigue loading: (**a**) transverse distribution; (**b**) longitudinal distribution.

Figure 12 illustrates the variation in strain amplitude of the bottom concrete subjected to fatigue loading. During fatigue loading, the non-uniform propagation of cracks led to significant variations in stress distribution within the bottom concrete at the mid-span. As depicted in Figure 12a, the bottom concrete strain of PC-HL gradually decreased. For specimens PC-LH, the C21 strain exceeded 140 με, and the strain gauge experienced failure after 0.2 M load cycles. In contrast, the strain value at C22, C23, and C24 remained relatively stable, exhibiting only minor decreases during the initial stages of fatigue loading. As shown in Figure 12, the concrete strain at mid-span was lower than that at the 1/4-span and 3/8-span, which could be attributed to the crack concentration at mid-span during the loading process. For PC-HL, the maximum strain value occurred at the 1/4-span, with its strain gauge failure after 1.8 M load cycles. For PC-LH, the highest strain was observed at the 3/8-span, and the strain gauge experienced failure after 0.05 M load cycles.

**Figure 13:**
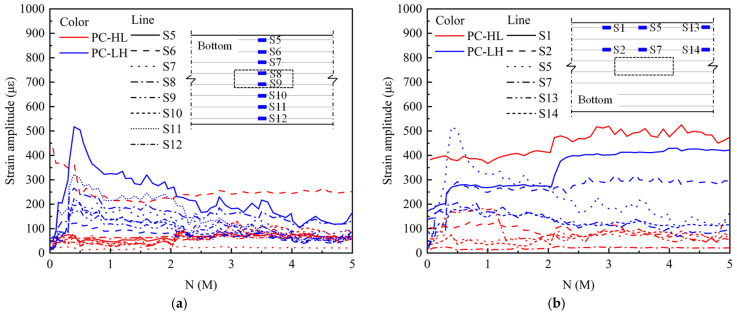
Strain amplitude of bottom rebar under fatigue loading: (**a**) transverse distribution; (**b**) longitudinal distribution.

Figure 13 depicts the variation in strain amplitude of the bottom rebar under fatigue loading. Figure 13a reveals that the strain of rebar in the PC slab exhibited an outer-to-inner decreasing gradient. For PC-LH, the rebar strain initially increased and subsequently decreased as the number of load cycles increased. PC-LH exhibited significant deformation during the initial stage of fatigue loading, which resulted in increased stress on the rebar. As loading progressed, crack initiation and propagation at the bottom of the slab caused localized increases in rebar stress near the cracks, whereas the strain of the rebar in non-cracked regions decreased. PC-HL occurred significant static damage prior to fatigue loading, resulting in a larger strain in the rebar during the initial stages of fatigue loading. However, the subsequent crack propagation reduced the rebar strain. When the load cycles exceeded 1 M, the strain variation stabilized. As illustrated in Figure 13b, the strain of S1 at 3/8-span was notably higher compared to other positions. The trend in the strain variation in S1 closely mirrors that of the displacement amplitude, suggesting that S1 was located proximal to the crack. Consequently, S1 can effectively capture and represent the force variation trend experienced by the bottom rebar during the fatigue loading.

## 4. Numerical Analysis

### 4.1. Finite-Element Model

**Figure 14:**
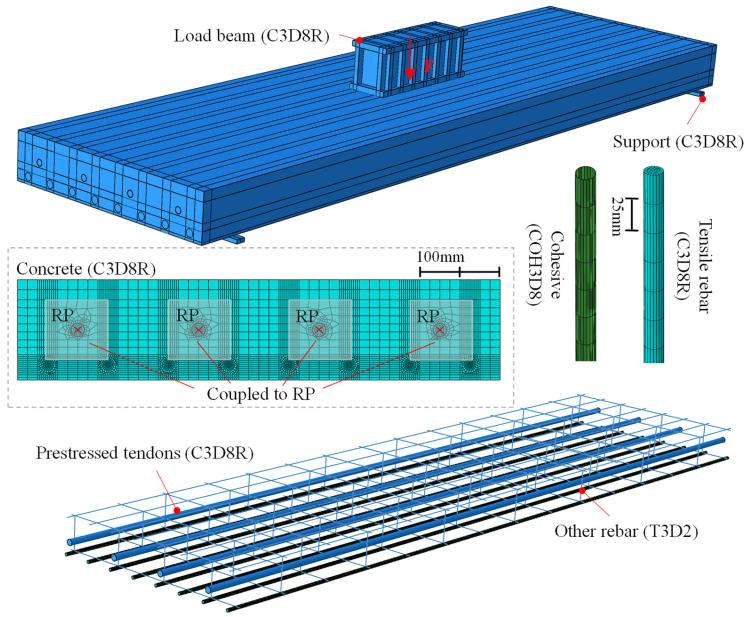
Finite-element model.

A finite-element model was established using general finite-element software ABAQUS2020 [29]. As depicted in Figure 14, the solid element C3D8R was employed to simulate concrete, bottom rebars, support, loading beam, and prestressed tendons, while other rebars were modeled with the T3D2 truss elements. The interaction between the loading beam and the slab was established using the “contact” command, which defined normal contact as hard and tangential contact as frictional, with a coefficient of friction set to 0.5 [30]. Constraints for other rebars within the concrete were implemented using the “embedded” command. The interaction between the bottom rebars and the concrete was simulated using zero-thickness cohesive elements (COH3D8). The interaction between the slab and the support was achieved using the “tie” command. The anchorage condition between the prestressed tendons and the slab was simulated using the “coupling” command, and the prestress was induced with the cooling method. To simulate the anchorage of prestressed tendons, all degrees of freedom of the cross-section at both ends of the prestressed tendons and the corresponding anchorage zones on the concrete were coupled to reference points. Prestress was applied using the thermal cooling method, in which the required temperature change *T* was calculated as *T* = *σ*/(*E*·*α*), with the coefficient of thermal expansion *α* taken as 1.2 × 10^−5^. A reference point was created on the upper surface of the loading beam for the application of loads.

**Figure 15:**
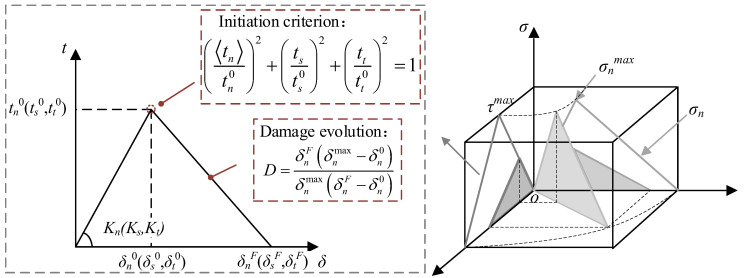
Bond–slip constitutive relationship.

The concrete damage plasticity (CDP) model was utilized to simulate the nonlinear behavior of concrete. The dilation angle, eccentricity, *f_b_*_0_/*f_c_*_0_, *K*, and viscosity parameter were selected as 30°, 0.1, 1.16, 0.67, and 0.0005, respectively [29,31], which had been validated in previous numerical simulations of concrete slab, and the mesh size and element type adopted in this study were consistent with those in previous work [6,7,27]. The axial compressive strength, elastic modulus, and axial tensile strength of concrete were determined to be 50.6 MPa, 30.8 GPa, and 2.6 MPa. The compressive and tensile constitutive curves of concrete were determined using Eurocode 2, 1992-1-1 [3], and the crack width *ω_c_*, representing the complete release of stress in the tensile constitutive curves, was selected as 0.097 mm. A trilinear model was employed to simulate the deformation behavior of rebar and steel plate, and the yield stress of the rebar, prestressed tendons, and steel plate was 484 MPa, 785 MPa, and 355 MPa, respectively. The bond parameters for rebar and concrete are presented in Table 2, where *t_n_*^0^ = *f_t_*, *√2t_n_*^0^ = *t_s_*^0^ = *t_t_*^0^, 10*G_n_* = *G_s_* = *G_t_* [32], *δ_n_^F^* = 0.1 mm. These parameters had been validated through test results reported in the literature [32], including rebar–concrete interface bond test and flexural test on reinforced concrete-beam specimens. The bilinear separation model and the quadratic stress criterion were adopted to simulate the bond damage behavior of rebar and concrete [33], as shown in Figure 15, where *K*, *t*, *δ*, and *G* represent the bond stiffness, the traction stress, the separation, and the fracture energy, respectively. Subscripts *n*, *s*, and *t* indicate that the normal, first and second tangential directions of the bond interface, respectively. Superscripts 0 and *F* indicate that the variable corresponds to the peak traction stress and the ultimate failure, respectively, and the symbol <> is the Macauley bracket.

### 4.2. Model Validation

**Figure 16:**
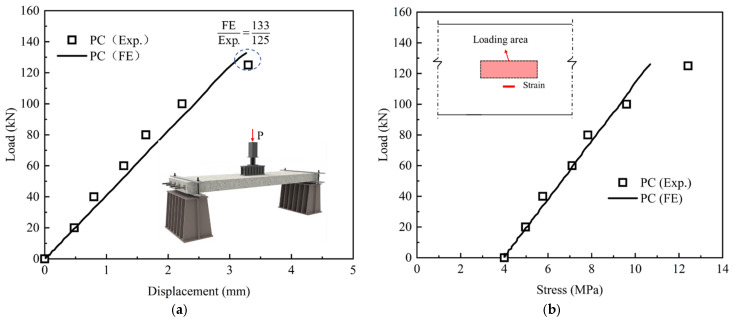
Comparison of test and numerical results: (**a**) load–displacement; (**b**) load–stress.

Figure 16 compares the load–displacement relations and load–strain relations from numerical calculation to the test results of PC-HL under the first static cyclic loading. The numerical results were in close agreement with the test results, thereby validating the reliability of the finite-element model.

### 4.3. Parametric Analysis of Finite-Element Model

**Figure 17:**
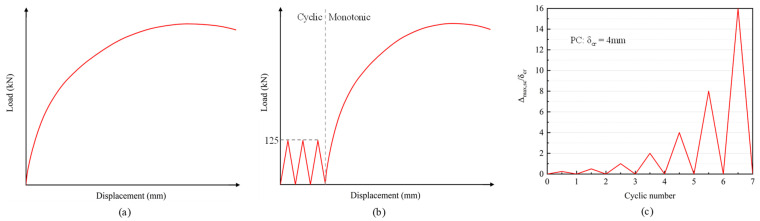
Loading program: (**a**) monotonic (M); (**b**) cyclic and monotonic (F); (**c**) cyclic (C).

A parametric analysis was conducted to discuss the mechanical deformation behavior, and the variable parameter is shown in Table 3. The number following PC indicates the prestress level, and the letters following the hyphen specify the loading methods: monotonic displacement loading (M), static cyclic loading followed by monotonic displacement loading (F), and cyclic displacement loading (C). For cyclic displacement loading, the maximum displacement value (*δ_max_*) was expressed as a multiple of the cracking displacement (*δ_cr_*). The loading schematic is illustrated in Figure 17.

#### 4.3.1. Load–Displacement Curves

**Figure 18:**
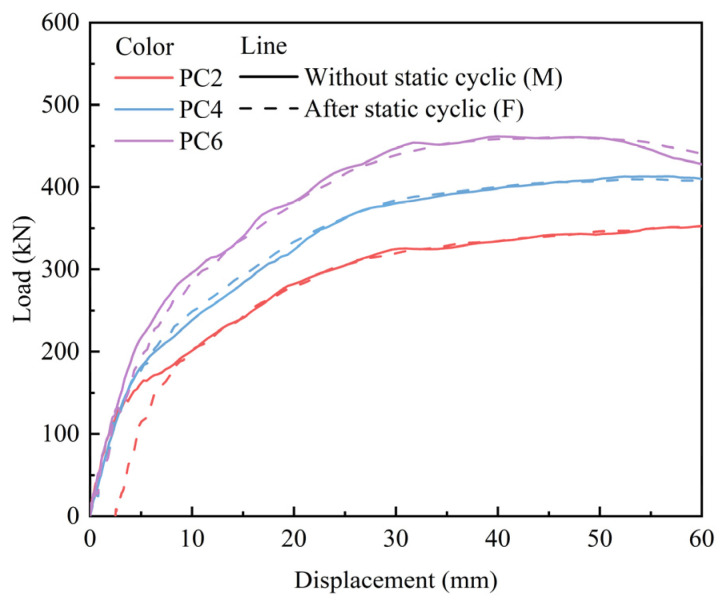
Load–displacement curves.

Figure 18 depicts the load–displacement curve under monotonic displacement loading and static cyclic loading followed by monotonic displacement loading. Applying prestress significantly enhanced the ultimate bearing capacity and ductility of the slab. Compared with the slab subjected to monotonic displacement loading, those subjected to static cyclic loading followed by monotonic displacement loading exhibited varying degrees of residual displacement during the monotonic loading stage, and the magnitude of residual displacement decreased as the prestress value increased. Notably, the residual displacement for specimens PC4 and PC6 approached zero. This suggests that the application of prestress significantly mitigates the damage induced by static cyclic loading on the slab.

#### 4.3.2. Evolution of Concrete Stress

**Figure 19:**
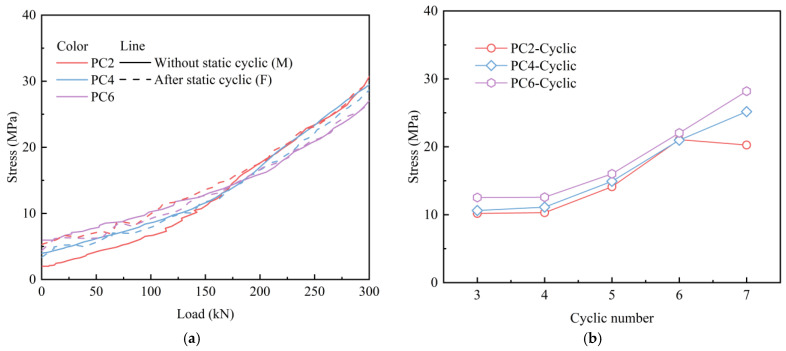
Stress variation in the top concrete: (**a**) monotonic loading; (**b**) cyclic loading.

Figure 19 shows the stress variation in the top concrete at the mid-span of the slab. The stress variation trend in the top concrete under monotonic displacement loading was similar to that observed under static cyclic loading followed by monotonic displacement loading. The stress curves of concrete for PC4 and PC6 specimens exhibited similar patterns under different loading conditions. For PC2 specimens, when the load was below 150–200 kN, the concrete stress in specimens subjected to static cyclic loading initially exceeded that of specimens under monotonic displacement loading. When the load exceeded 200 kN, the stresses exhibited similar characteristics. Figure 19b depicts the stress variation in the top concrete under cyclic displacement loading when the load reached 125 kN. The prestress resulted in high concrete stress in the PC slab, which increased significantly with the number of load cycles. Additionally, the stress of top concrete in PC2 exhibited a decline at the end of the cyclic loading.

#### 4.3.3. Evolution of Rebar Stress

**Figure 20:**
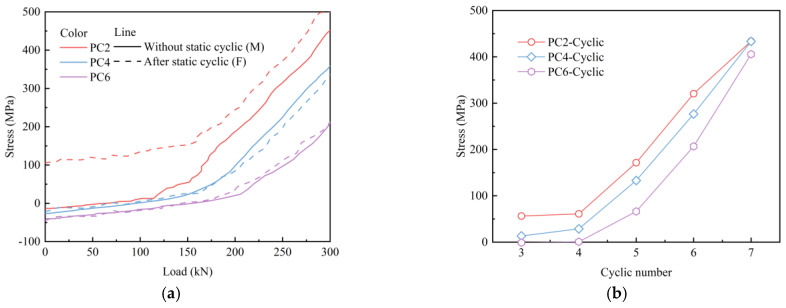
Stress variation in the bottom rebar: (**a**) monotonic loading; (**b**) cyclic loading.

Figure 20 illustrates the stress variation in the bottom rebar at the mid-span of the slab. As shown in Figure 20a, the application of prestress induced compressive stress on the bottom rebar of PC slabs. However, as the load increases, the stress in the bottom rebar rose significantly, indicating that cracks initiated at the bottom of the slab, causing the bottom concrete to exit from the load-bearing state and triggering stress redistribution. When the slab was initially subjected to static cyclic loading, the bottom rebar stress in PC2 specimens increased significantly, while no significant stress variation was observed in PC4 and PC6 specimens. Figure 20b shows the stress variation in the bottom rebar under cyclic displacement loading when the load reached 125 kN. Compared to PC2, the PC4 and PC6 slabs exhibited lower stress levels in the bottom rebar under the same number of load cycles, indicating that prestress significantly improved the stress distribution in the tensile zone of the slab.

#### 4.3.4. Rebar–Concrete Bond Damage

**Figure 21:**
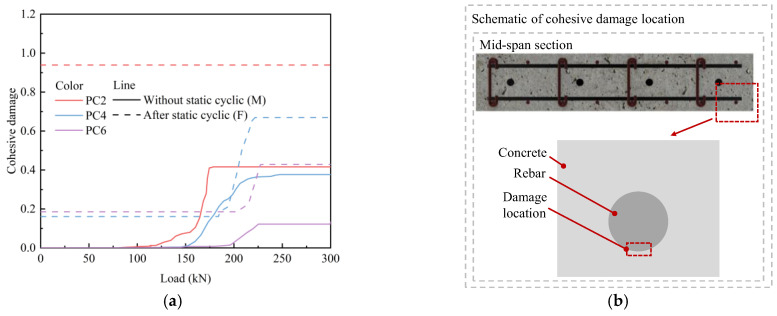
Monotonic load-bond damage curve: (**a**) damage curve; (**b**) location for damage data extraction.

Figure 21 depicts the bond damage between the bottom of the mid-span rebar and concrete under monotonic displacement loading, and static cyclic loading followed by monotonic displacement loading. Applying prestress significantly improved the bond performance between rebar and concrete, and the extent of bond damage between the rebar and concrete diminished as the prestress level increased. After the slabs were subjected to static cyclic loading, the bond damage between the rebar and concrete increased substantially. For PC2 specimens, the rebar–concrete bond approached complete degradation, whereas the increase in bond damage for the PC4 and PC6 specimens remains relatively minor.

### 4.4. Performance Enhancement Mechanism

**Figure 22:**
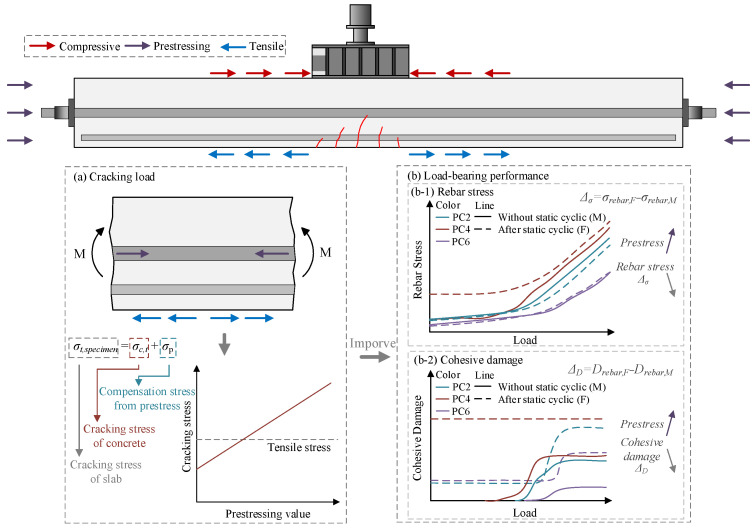
Force analysis of the PC slab.

Figure 22 presents a schematic diagram illustrating the force analysis of the PC slab. Applying prestress could effectively counteract the cracking stress in concrete according to the stress analysis of tensile rebar in the concrete slab under cyclic displacement loading, delaying the onset of cracking in the bottom, thereby reducing the stress concentration in the tensile rebar. This improvement enhanced mechanical deformation behaviors, including the redistribution of rebar stresses and bond damage between rebar and concrete, resulting from cracking. Consequently, the fatigue life of the PC slab was significantly extended. Furthermore, prestress could reduce the adverse effects of initial static damage on both the tensile rebar stress and the interfacial bond stress of rebar and concrete, thus enhancing the structural performance of the slab following prior damage.

## 5. Theoretical Analysis

### 5.1. Fatigue Characterization

#### 5.1.1. Compression Zone Concrete

During the fatigue loading process, the concrete damage and the reduction in elastic modulus accumulate with load cycles. The fatigue damage of concrete is characterized by evaluating the development of residual strain and changes in elastic modulus. The residual strain of concrete was determined using Equation (1) [34]:

(1) $$lg\varepsilon_{c,n}=t\cdot lgn+a_{\sigma }$$

where *t* is a constant with a value of 0.1148, *n* represents the number of load cycles, and *a_σ_* is a parameter associated with fatigue stress, determined using Equation (2) [34]:

(2) $$a_{\sigma }=0.0337\sigma_{c,\max}-5.34115$$

where *σ_c,max_* represents the maximum compressive stress of concrete in the compression zone.

The elastic modulus of concrete under fatigue loading could be determined using the residual strain of concrete, as presented in Equation (3) [26]:

(3) $$E_{c,n}=\frac{\sigma_{c,\max}}{\sigma_{c,\max}/E_{c}+\varepsilon_{c,n}}$$

where *E_c,n_* represents the elastic modulus after *n* load cycles; *E_c_* denotes the initial elastic modulus of concrete.

#### 5.1.2. Tensile Rebar and Prestressed Tendons

Cracks in the tensile rebar initiate and propagate from a random material defect located at the rib of the rebar under fatigue load. Furthermore, the rebar–concrete bond is a critical factor that enables tensile rebar to effectively bear tensile stress [35], while fatigue loading leads to degradation of the rebar–concrete bond, consequently diminishing the effectiveness of the tensile rebar. The cumulative influence of the above factors results in a reduction in the effective cross-sectional area of the tensile rebar, which is a virtual change and does not indicate an actual decrease in the cross-sectional area of the tensile rebar. Hence, the residual cross-sectional area method for rebar was utilized to quantify the fatigue damage, as presented in Equation (4) [36]:

(4) $$A_{s,b,n}=A_{s,b}\cdot (1-\frac{n}{N_{s}}(1-\frac{\sigma_{s,max,f}}{f_{y}}))$$

The reduction in the effective cross-sectional area of the prestressed tendons is primarily attributed to crack propagation initiated from the material defect, as indicated in Equation (5):

(5) $$A_{p,n}=A_{p}\cdot (1-\frac{n}{N_{p}}(1-\frac{\sigma_{p,\max,f}}{f_{pt}}))$$

where *A_s,b,n_* and *A_p,n_* represent the effective cross-sectional areas of the tensile rebar and prestressed tendons, respectively, after *n* load cycles; *A_s,b_* and *A_p_* denote the initial cross-sectional areas of the tensile rebar and prestressed tendons, respectively; *N_s_* and *N_p_* indicate the number of load cycles that the tensile rebar and prestressed tendons can withstand; *σ_s,max,f_* and *σ_p,max,f_* are the maximum stresses experienced by the tensile rebar and prestressed tendons under fatigue loading; *f_y_* represents the yield strength of the tensile rebar; *f_pt_* denotes the ultimate strength of the prestressed tendons, which was specified as 930 MPa.

The permissible load cycles for tensile rebar and prestressed tendons were determined using Equations (6) and (7):

(6) lgN=b−k⋅lgΔσ

(7) $$\Delta \sigma =\sigma_{max,f}-\sigma_{min,f}$$

where *b* and *k* are constants with values of 14.8 and 3.8 [37], respectively; *σ_max,f_* and *σ_min,f_* represent the maximum and minimum stress of the rebars and prestressed tendons, respectively, under different cycle numbers.

### 5.2. Failure Criterion

The fatigue damage of a concrete slab is a progressive and irreversible process. As the number of load cycles increases, internal cracks within the concrete continue to propagate, leading to a continuous increase in residual strain in the compression zone. The failure criterion for concrete was presented in Equation (8) [38]:

(8) $$\varepsilon_{c,n}\geq 0.4\cdot f_{c}/E_{c}$$

where *f_c_* denotes the axial compressive strength of concrete.

Stress was utilized as the criterion for assessing fatigue failure in tensile rebar and prestressed tendons. Fatigue failure is initiated when the stress surpasses either the yield strength of the tensile rebar or the ultimate tensile strength of the prestressed tendons, as indicated in Equation (9) [24]:

(9) $$\sigma_{s,max,f}>f_{y},\sigma_{p,max,f}>f_{pt}$$

### 5.3. Force Analysis

**Figure 23:**
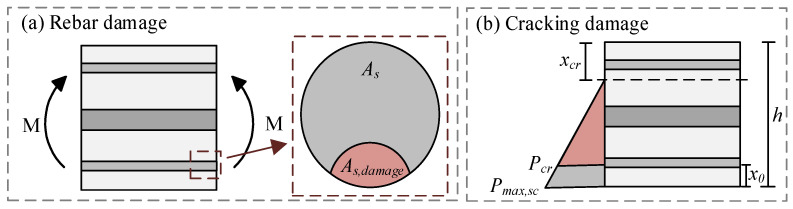
Damage indication: (**a**) rebar damage; (**b**) cracking damage.

The influence of initial damage on the fatigue performance was investigated by initially applying static cyclic loading followed by fatigue loading, and the method of reducing the effective height of the concrete in the bridge slab was employed to simulate the initial damage induced by heavy vehicular loads. As depicted in Figure 23, the initial damage to the bridge slab primarily included rebar deterioration and crack formation. As discussed in Section 5.1, the stress induced in the rebar by the external load led to a reduction in the effective cross-sectional area of the rebar; consequently, the reduction in the effective cross-sectional area was utilized as an indicator of rebar damage. The cracking of the concrete for bridge slabs is primarily attributed to external loads; hence, the extent of cracking damage in the bridge slab could be characterized by the ratio of the cracking load to the applied external load. The equivalent damage height of the bridge slab was determined using Equation (10).

(10) $$x_{0}=\left\{\begin{array}{cc} max(\frac{A_{s,b,sc}}{A_{s,b}}h,(1-\frac{P_{cr}}{P_{max,sc}})\cdot (h-x_{cr}))\;\;\;\;P_{max,f}\leq P_{max,sc} & \\ \left(\frac{P_{max,f}}{P_{max,sc}})\cdot max(\frac{A_{s,b,sc}}{A_{s,b}}h,(1-\frac{P_{cr}}{P_{max,sc}})\cdot (h-x_{cr})\right)\;\;\;\;P_{max,f}>P_{max,sc} & \end{array}\right.$$

where *x*_0_ represents the equivalent damage height of the concrete at the slab section; *h* denotes the height of the slab; *P_max,sc_* signifies the maximum value of the static cyclic load; *P_max,f_* indicates the maximum value of the fatigue load; *P_cr_* is the cracking load of the PC slab, which was calculated using Equation (11). Additionally, *x_cr_* represents the height of the compression zone when the PC slab is fully cracked, which was determined based on the analysis of the fully cracked cross-section. *A_s,b_* represents the area of the tensile rebar; Lastly, *A_s,b,sc_* refers to the equivalent cross-sectional area of the bottom rebar after static cyclic loading, which was computed using Equations (12) and (13).

(11) $$P_{cr}=\frac{4B_{sc}}{l}\cdot \frac{f_{t}+\sigma_{c,p}}{\left(h-x_{sc}\right)\cdot E_{c}}$$

where *l* denotes the effective span of the PC slab; *B_sc_* is the section stiffness under static cyclic loading determined by cross-section analysis; *x_sc_* denotes the height of the compression zone under static cyclic loading calculated according to cross-section analysis; *f_t_* refers to the tensile strength of concrete as specified by fib-Model Code 2010 [39]; and *σ_c,p_* indicates the prestress stress.

(12) $$A_{s,b,sc}=A_{s,b}\cdot (1-\frac{\sigma_{s}}{f_{y}})$$

(13) $$\sigma_{s}=\sigma_{s,max,sc}-\sigma_{s,p}$$

where *σ_s_* represents the stress of tensile rebars under static cyclic loading; *σ_s,p_* denotes the stress of tensile rebars induced by prestress; and *σ_s,max,sc_* signifies the stress of tensile rebars at the maximum static load, as calculated using Equations (14) and (15):

(14) $$\sigma_{s,max,sc}=E_{s}\kappa_{sc}\cdot (h-x_{sc}-a_{b})$$

(15) $$\kappa_{sc}=\frac{M_{sc}}{B_{sc}}$$

where *κ_sc_* represents the curvature induced by static cyclic loading; and *M_sc_* denotes the section bending moment under static cyclic loading.

The mid-span displacement under static cyclic loading was determined using the principle of virtual work [40], as presented in Equation (16):

(16) $$\Delta_{sc}=\sum \int \frac{M\bar{M}}{B_{sc}}ds+\sum \int \frac{kV\bar{V}}{G_{sc}A_{0}}ds$$

where Δ*_sc_* signifies the mid-span displacement under static cyclic loading; *G_sc_* denotes the shear modulus of concrete under static cyclic loading, which is 0.4*E_c_*, *A*_0_ represents the cross-section area of slab.

To account for the influence of top concrete damage, tensile rebar damage, prestressed tendon damage during static cyclic loading, and both static and fatigue loads on the deformation of the slab, a damage deformation coefficient *φ* was introduced, as presented in Equation (17):

(17) $$\varphi =\left\{\begin{array}{cc} {\left(\frac{\sigma_{c}}{0.4f_{c}}+\frac{\sigma_{s}}{f_{y}}+\frac{\sigma_{p}}{f_{pt}}+\frac{P_{cr}-P_{max,sc}}{P_{max,sc}}\right)}^{m}+\frac{P_{max,f}-P_{cr}}{P_{cr}}\;\;\;\;P_{max,sc}<P_{cr} & \\ {\left(\frac{\sigma_{c}}{0.4f_{c}}+\frac{\sigma_{s}}{f_{y}}+\frac{\sigma_{p}}{f_{pt}}\right)}^{m}+\frac{P_{max,f}-P_{max,sc}}{P_{max,sc}}\;\;\;\;P_{max,sc}\geq P_{cr} & \end{array}\right.$$

where *σ_c_* represents the stress experienced by the top concrete under static cyclic loading; *σ_p_* denotes the stress in the prestressed tendons under the static cyclic loading; *m* signifies the number of static cyclic load cycles.

The stress in the top concrete and the stress in the prestressed tendons were determined using Equations (18) and (19).

(18) $$\sigma_{c}=E_{c}\kappa_{sc}x_{sc}+\sigma_{c,p}$$

(19) $$\sigma_{p}=E_{p}\kappa_{sc}\cdot (\frac{h}{2}-x_{sc})+\sigma_{p,p}$$

where *σ_c,p_* represents the stress in the top concrete induced by prestress; *σ_p,p_* denotes the stress in the prestressed tendons resulting from prestress, *E_p_* is referred to the elastic modulus of the prestressed tendons.

**Figure 24:**
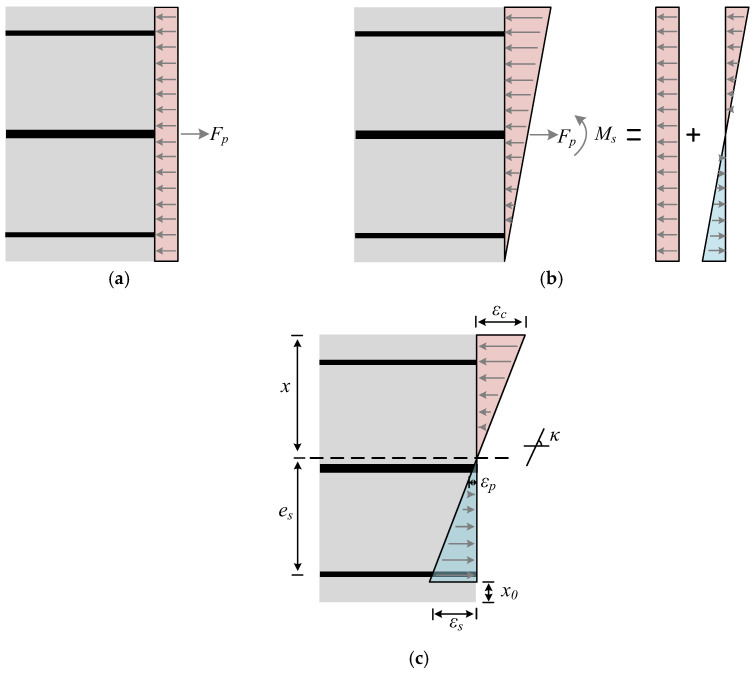
Force and strain distribution in the PC slab section: (**a**) effective prestress; (**b**) concrete stress at bottom edge equals zero; (**c**) fatigue loading.

During the fatigue-loading process, the force behavior in the PC slab could be categorized into three stages: (a) the effective prestress stage, (b) the initial load application stage where the stress at the bottom edge of the slab became zero, and (c) the fatigue-loading stage, as illustrated in Figure 24. The following assumptions were made during the analysis:

(1) The PC slab that adheres to the plane section remains a plane assumption.
(2) The prestress value was maintained at a constant level throughout the fatigue cycle.
(3) The influence of crack width on the deformation of the slab is ignored.

For stage (a), the prestress was transmitted to the cross-section of the slab via the prestressed tendons.

For stage (b), the concrete stress at the bottom edge of the slab reached zero. As the external load gradually increased, the prestress at the bottom of the PC slab was reduced to zero, with the neutral axis positioned at the bottom edge of the slab. The curvature could be determined using Equation (20), allowing for the calculation of stress in the concrete, tensile rebar, and prestressed tendons.

(20) $$\kappa_{0}=\frac{M_{s}}{B_{0}}$$

where *κ*_0_ represents the section curvature when the concrete stress at the bottom edge was zero, *M_s_* denotes the section bending moment under fatigue loading, and *B*_0_ signifies the section stiffness when the tensile stress of the concrete at the bottom edge was zero.

At this stage, the stress in the top concrete, the tensile rebar, and the prestressed tendons were calculated separately using Equations (21)–(23).

(21) $$\sigma_{c2}=E_{c}\kappa_{0}h$$

(22) $$\sigma_{s2}=E_{s}\kappa_{0}a_{b}$$

(23) $$\sigma_{p2}=E_{p}\kappa_{0}\frac{h}{2}$$

where *σ_c_*_2_, *σ_s_*_2_, and *σ_p_*_2_ represent the stress in the top concrete, the tensile rebar, and the prestressed tendons, respectively, when the edge stress was zero; *E_s_* refers to the elastic modulus of the rebar.

For stage (c), the cross-section of the PC slab adhered to the plane section assumption. Consequently, the stress in the top concrete, tensile rebar, and the prestressed tendons could be described by Equations (24)–(30).

(24) $$\sigma_{c,max,f}=\sigma_{c,p}+\sigma_{c2}+\sigma_{c,n}$$

(25) $$\sigma_{c,n}=E_{c,n}\kappa_{n}x_{n}$$

(26) $$\sigma_{s,max,f}=-\sigma_{s1}+\sigma_{s2}+\sigma_{s,n}$$

(27) $$\sigma_{s,n}=E_{s}\kappa_{n}\cdot (h-x_{n}-a_{b})$$

(28) $$\sigma_{p,max,f}=\sigma_{p,p}+\sigma_{p2}+\sigma_{p,n}$$

(29) $$\sigma_{p,n}=E_{p}\kappa_{n}\cdot (\frac{h}{2}-x_{n})$$

(30) $$\kappa_{n}=\frac{M_{s}}{B_{n}}$$

where *σ_c,max_._f_* denotes the maximum stress of top concrete at *n* load cycles; *σ_c,n_*, *σ_s,n_*, and *σ_p,n_* represent the stress in the top concrete, tensile rebar, and the prestressed tendons, respectively, during the *n* load cycles of the fatigue-load cycles; *x_n_* denotes the height of the concrete compression zone at *n* load cycles. *B_n_* represents the section stiffness after *n* load cycles; *κ_n_* represents the sectional curvature after *n* load cycles.

The mid-span displacement of the PC slab under fatigue loading was determined using the principle of virtual work [35], incorporating the influence of initial damage before fatigue loading on the fatigue deformation of the PC slab, as expressed in Equation (31).

(31) $$\Delta_{f,n}=\varphi (\sum \int \frac{M\bar{M}}{B_{n}}ds+\sum \int \frac{kV\bar{V}}{G_{n}A_{0}}ds)$$

where *G_n_* represents the shear modulus of concrete subjected to *n* load cycles, which is 0.4*E_c,n_*, and the factor *k* is set to 1.2.

**Figure 25:**
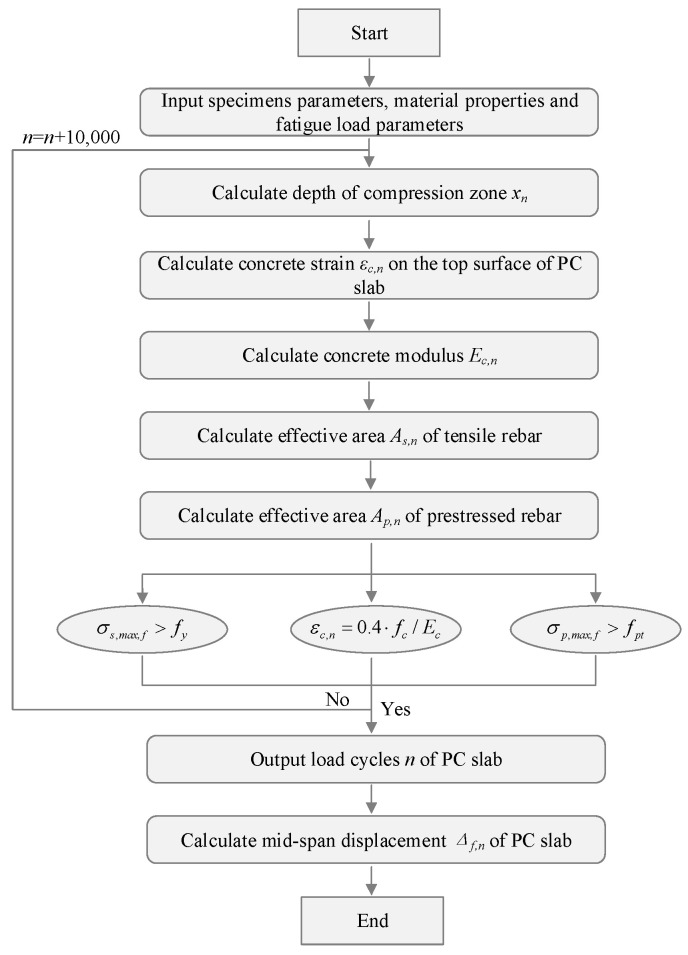
Analysis process for the fatigue behavior of PC slabs.

The fatigue performance evaluation method for the PC slab was illustrated in Figure 25.

### 5.4. Model Verification

Table 4 compares the calculated static cyclic displacements with test results, with errors less than 10%, demonstrating that the analytical model for deformation of the PC slab under static cyclic loading was accurate.

**Figure 26:**
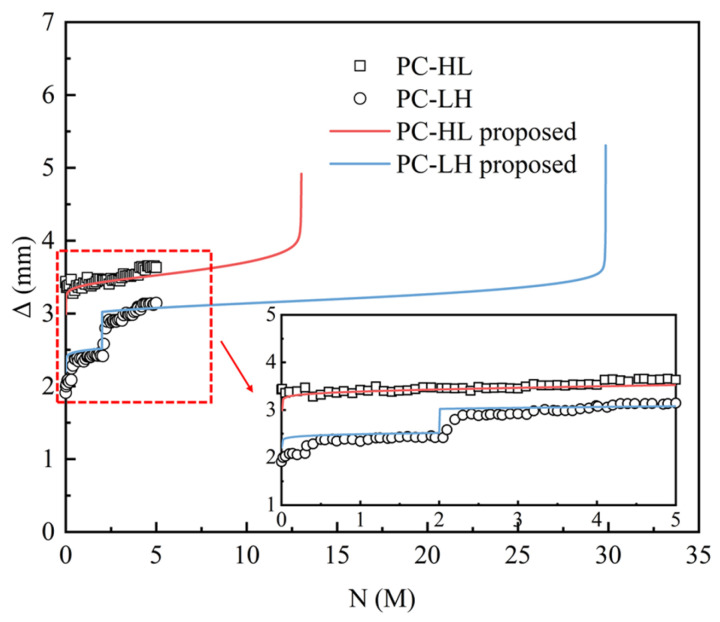
Verification of the performance evaluation method.

Figure 26 compares the results of the fatigue performance evaluation method, which incorporates initial damage, against the test results. The proposed method accurately characterized the displacement variation in the PC slab during the initial 5 M load cycles under constant and variable amplitude loading conditions. This demonstrates that the method could precisely predict the fatigue deformation behavior of the PC slab. Furthermore, the loadable cycles for the specimens PC-HL and PC-LH were 13.04 M and 29.85 M, respectively. Both types of slabs exhibited a failure mode characterized by the fracture of the tensile rebar.

### 5.5. Parametric Analysis

A parametric analysis was conducted to investigate the influence of prestress values on the static and fatigue performance of the slab. Different prestress values were applied by adjusting the tensioning stress (S) and the cross-sectional area of the prestressed tendons (A). While the tensioning stress of tendons was varied, the dimensions of the slab and the prestress layout were identical to those of the test models, with the tensioning stress varying from 0 to 90% of the ultimate strength of the prestressing tendons. While the tendon area was varied, the tensioning stress was kept at 75% of the ultimate tensile strength, and the dimensions of the slab and the position of the prestressed tendons were consistent with the test models. Furthermore, the static and fatigue-loading procedures were identical to those applied to specimen PC-HL, as outlined in Table 5.

**Figure 27:**
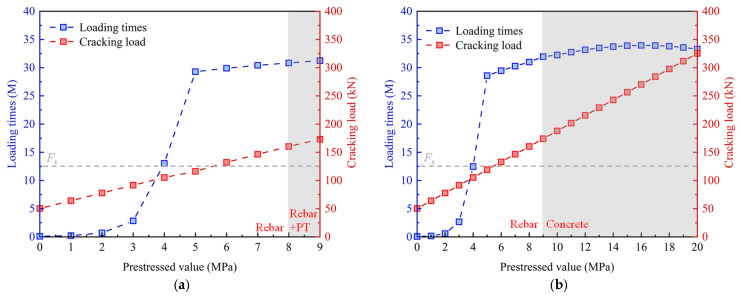
Cracking load and the number of load cycles under various prestress: (**a**) variation in tensioning stress; (**b**) variation in prestressed tendons area.

Figure 27 illustrates the cracking load and the number of load cycles for the PC slab under varying levels of prestress. Applying prestress could significantly enhance the cracking load and increase the number of load cycles that the slab can withstand. Furthermore, when the prestress value exceeded 5 MPa, leading to a cracking load surpassing 90% of the maximum static load, the rate of increase in the number of load cycles became relatively slower. Figure 27a indicates that when the prestress value exceeded 8 MPa, the prestressed tendons fractured upon fatigue failure of the slab. At this point, the ratio of the tensioning stress to the ultimate strength of the prestressed tendons surpasses 80%. As depicted in Figure 27b, when the tensioning stress was maintained at 75% of the ultimate strength, the prestressed tendons remained intact even as the slab experienced fatigue failure. However, if the prestress value exceeded 9 MPa, the failure mode of the PC slab shifts from rebar fracture to concrete failure.

## 6. Conclusions

To investigate the performance enhancement of the PC slab in composite bridges and evaluate its fatigue performance, two identical full-scale models of PC slabs were designed and tested, with the load amplitude being the main variable. The models were loaded with an initial static cyclic loading followed by fatigue loading, to simulate the damage of the slab after experiencing heavy vehicular and subsequently bearing regular traffic loads. The evolution of displacements and strains was analyzed. Refined finite-element models that incorporated the bond damage were developed to reveal the performance enhancement mechanism of PC slabs. Meanwhile, a fatigue performance-evaluation method that incorporates static cyclic damage was developed, and its accuracy was validated through test results. The following conclusions can be drawn:

(1) By comparing the test results of the PC slab under different static load cycles, it is evident that high static loads induce significant cracking in the slab, whereas cracks under low static loads remain in the initial stages. During subsequent fatigue loading, the slab subjected to low static loads exhibits smaller deformations and lower stresses in both concrete and rebar. Even if the upper limit of the fatigue load is increased to match the level of high static loads, the resulting deformation and stress levels remain relatively small. This indicates that damage caused by static cyclic loading has a substantial influence on the fatigue performance of the slab.
(2) Based on the numerical results, applying prestress can effectively mitigate crack initiation and propagation by compensating for tensile stresses at the bottom of the slab. This not only enhances the redistribution of rebar stress around cracks but also slows down the degradation of rebar–concrete bond, and the effectiveness becomes more pronounced with increasing prestress values, thereby reducing the influence of static cyclic damage on the fatigue performance of the slab.
(3) In the established method for evaluating the fatigue performance of PC slab, the concept of equivalent damage height was introduced to represent initial damage, and a damage deformation parameter was proposed to account for the influence of initial damage on fatigue deformation, and the performance degradation of both the concrete and rebar in the slab under fatigue load was characterized independently. Comparison with test data demonstrates that the evaluation method accurately reflects the fatigue deformation of the PC slab, with an average error margin below 10%, and reliably predicts the fatigue life and failure mode.
(4) The parametric analysis based on the evaluation method reveals that when the tensioning stress of the prestressed tendons exceeds 80% of their ultimate strength, the prestressed tendons fracture upon fatigue failure of the PC slab. Conversely, maintaining the tensioning stress at 75% of the ultimate strength prevents such fractures. Furthermore, increasing the prestressed value exceeds 9 MPa by altering the area of the prestressed tendons shifts the failure mode of the PC slab from rebar fracture to concrete failure.

## Figures and Tables

**Table 1:** Model parameters.

ID	Static Stage	Fatigue Stage I (0–2 M Cycle)		Fatigue Stage II (2–5 M Cycle)	
	*F_s_* (kN)	*F_min_* (kN)	*F_max_* (kN)	*F_min_* (kN)	*F_max_* (kN)
PC-HL	125	40	100	40	100
PC-LH	100	40	100	65	125

**Table 2:** Bond parameters of rebar and concrete.

*K*_n_ (N/mm^3^)	*K*_s_, *K_t_* (N/mm^3^)	*t*_n_^0^ (MPa)	*t*_s_^0^, *t*_t_^0^ (MPa)	*G*_n_ (N/mm)	*G*_s_, *G_t_* (N/mm)
40,690	20,410	2.60	3.68	0.13	1.30

**Table 3:** Variable parameter in finite-element model.

ID	Prestress Value (MPa)	Top Rebar		Bottom Rebar		Loading Methods
		Diameter (mm)	Quantity	Diameter (mm)	Quantity	
PC2-M	2	16	8	16	8	Monotonic
PC2-F	2	16	8	16	8	Cyclic + Monotonic
PC2-C	2	16	8	16	8	Cyclic
PC4-M	4	16	8	16	8	Monotonic
PC4-F	4	16	8	16	8	Cyclic + Monotonic
PC4-C	4	16	8	16	8	Cyclic
PC6-M	6	16	8	16	8	Monotonic
PC6-F	6	16	8	16	8	Cyclic + Monotonic
PC6-C	6	16	8	16	8	Cyclic

**Table 4:** Verification of maximum displacement under static cyclic loading.

ID	Cycle	Test Result (mm)	Calculation Result (mm)	Error (%)
PC-HL	1	3.342	3.188	4.61
	2	3.356	3.199	4.68
	3	3.391	3.200	5.63
PC-LH	1	2.346	2.551	8.04
	2	2.418	2.557	5.75
	3	2.415	2.558	5.92

**Table 5:** Variable parameter.

ID	*F_min_* (kN)	*F_min_* (kN)	*F_max_* (kN)	Prestress (MPa)	Tensioning Stress (MPa)	Stress/Ultimate Strength (%)	Area (mm^2^)	*x*_0_ (mm)
PC-S0	125	40	100	0	0.0	0	3216.9	110.45
PC-S1				1	93.3	10		90.05
PC-S2				2	186.5	20		69.79
PC-S3				3	279.8	30		49.39
PC-S4				4	373.0	40		29.13
PC-S5				5	466.3	50		18.53
PC-S6				6	559.5	60		18.01
PC-S7				7	652.8	70		17.49
PC-S8				8	746.0	80		16.97
PC-S9				9	839.3	90		16.45
PC-A0	125	40	100	0	697.5	75	0.0	110.45
PC-A1				1			430.1	90.20
PC-A2				2			860.2	69.79
PC-A3				3			1290.3	49.53
PC-A4				4			1720.4	29.13
PC-A5				5			2150.5	18.53
PC-A6				6			2580.7	18.01
PC-A7				7			3010.8	17.49
PC-A8				8			3440.9	16.97
PC-A9				9			3871.0	16.45
PC-A10				10			4301.1	15.93
PC-A11				11			4731.2	15.41
PC-A12				12			5161.3	14.89
PC-A13				13			5591.4	14.37
PC-A14				14			6021.5	13.85
PC-A15				15			6451.6	13.33
PC-A16				16			6881.7	12.81
PC-A17				17			7311.8	12.29
PC-A18				18			7741.9	11.77
PC-A19				19			8172.0	11.25
PC-A20				20			8602.2	10.73

## Data Availability

The original contributions presented in this study are included in the article. Further inquiries can be directed to the corresponding author.
